# 4-Anilino-3-nitro­benzonitrile

**DOI:** 10.1107/S1600536810043862

**Published:** 2010-11-24

**Authors:** Yong Wang, Kaiqing Fan, Chenghong Li, Changhua Ge

**Affiliations:** a School of Pharmaceutical and Chemical Engineering, Taizhou University, Linhai 317000, People’s Republic of China; b Agronomy Department, Jiangsu Polytechnic College of Agriculture and Forestry, Jurong 212400 Jiangsu, People’s Republic of China

## Abstract

In the title compound, C_13_H_9_N_3_O_2_, the aromatic rings are twisted with respect to each other, making a dihedral angle of 49.41 (9)°. The nitro group and the nitrile group are nearly in the plane of the benzonitrile ring, the largest deviation from the plane being 0.123 (1) Å. There is an intra­molecular N—H⋯O hydrogen bond forming an *S*(6) ring. Weak inter­molecular C—H⋯O hydrogen bonds link the mol­ecules into a chain parallel to the *c* axis. Futhermore, slipped π–π inter­actions between symmetry-related phenyl rings [centroid–centroid distance 3.808 (1) Å, inter­planar distance 3.544 (8) Å with an offset of 21.5°] stabilize the structure.

## Related literature

For the synthesis of the title compound, see: Schelz & Inst (1978 ▶). For related structures, see: McWilliam *et al.* (2001 ▶); Li, Liu *et al.* (2009 ▶); Li, Wu *et al.* (2009 ▶). For discussion of hydrogen bonding, see: Etter *et al.* (1990 ▶); Bernstein *et al.* (1995 ▶).
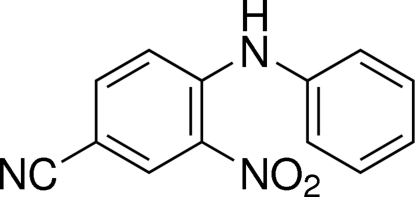


## Experimental

### 

#### Crystal data

C_13_H_9_N_3_O_2_
*M* *_r_* = 239.23
Monoclinic, 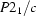

*a* = 14.066 (3) Å
*b* = 7.4290 (15) Å
*c* = 11.652 (2) Å
β = 109.04 (3)°
*V* = 1151.0 (4) Å^3^
*Z* = 4
Mo *K*α radiation
μ = 0.10 mm^−1^
*T* = 293 K
0.30 × 0.30 × 0.10 mm

#### Data collection

Enraf–Nonius CAD-4 diffractometer
Absorption correction: ψ scan (North *et al.*, 1968 ▶) *T*_min_ = 0.972, *T*_max_ = 0.990
4199 measured reflections
2082 independent reflections
1546 reflections with *I* > 2σ(*I*)
*R*_int_ = 0.030
3 standard reflections every 200 reflections  intensity decay: 1%

#### Refinement

*R*[*F* ^2^ > 2σ(*F* ^2^)] = 0.040
*wR*(*F* ^2^) = 0.107
*S* = 1.04
2082 reflections
163 parameters
H-atom parameters constrained
Δρ_max_ = 0.19 e Å^−3^
Δρ_min_ = −0.16 e Å^−3^

### 

Data collection: *CAD-4 Software* (Enraf–Nonius, 1989 ▶); cell refinement: *CAD-4 Software*; data reduction: *XCAD4* (Harms & Wocadlo, 1995 ▶); program(s) used to solve structure: *SHELXS97* (Sheldrick, 2008 ▶); program(s) used to refine structure: *SHELXL97* (Sheldrick, 2008 ▶); molecular graphics: *ORTEPIII* (Burnett & Johnson, 1996 ▶), *ORTEP-3 for Windows* (Farrugia, 1997 ▶) and *PLATON* (Spek, 2009 ▶); software used to prepare material for publication: *SHELXL97*.

## Supplementary Material

Crystal structure: contains datablocks global, I. DOI: 10.1107/S1600536810043862/dn2613sup1.cif

Structure factors: contains datablocks I. DOI: 10.1107/S1600536810043862/dn2613Isup2.hkl

Additional supplementary materials:  crystallographic information; 3D view; checkCIF report

## Figures and Tables

**Table 1:** Hydrogen-bond geometry (Å, °)

*D*—H⋯*A*	*D*—H	H⋯*A*	*D*⋯*A*	*D*—H⋯*A*
N1—H1⋯O1	0.86	1.96	2.6280 (18)	134
C3—H3*A*⋯O2^i^	0.93	2.59	3.478 (2)	159

Symmetry code: (i) 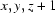.

**Fig. 1.**
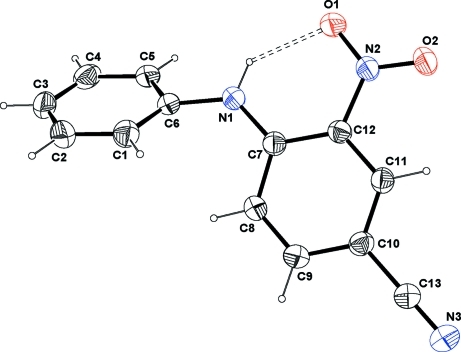
The molecular structure of (I), showing the atom-labeling scheme. Displacement ellipsoids are drawn at the 30% probability level. H atoms are represented as small spheres of arbitrary radii. Hydrogen bond is shown as dashed line.

**Fig. 2.**
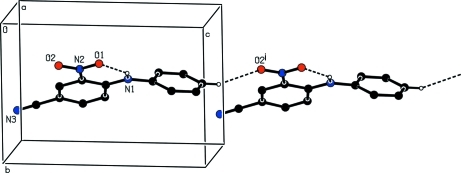
A partial packing view of (I) showing the infinite chain formed by C-H···O hydrogen bonds. H atoms not involved in hydrogen bondings have been omitted for clarity. [Symmetry code: (i) x, y, z+1]

## supplementary crystallographic information

### Comment

The molecule of the title compound is non planar, the two phenyl rings make a dihedral angle of 49.41 (9)°. The nitro and nitrile groups are nearly in the plane of the C7–C12 phenyl ring with the largest deviation being 0.123 (1) Å at O2 (Fig. 1). Bond lengths and bond angles agree with related structures recently reported (Li, Liu *et al.*, 2009; Li, Wu *et al.*, 2009; McWilliam *et al.*, 2001)

There is an intramolecular N-H···O hydrogen bond forming S(6) ring (Etter *et al.*, 1990; Bernstein *et al.*, 1995). A weak intermolecular C-H···O interactions link the molecule into a chain parallel to the c axis (Table 1, Fig. 2). Futhermore sliipest π-π interaction between symmetry related phenyl rings (symmetry code: (i) x,3/2-y,1/2+z) stabilize the structure (centroid to centroid 3.808 (1)Å, interplanar distance 3.544 (8)Å with an offset of 21.5° .

### Experimental

4-chloro-3-nitrobenzonitrile (4.0 g, 0.022 mol)was heat in 10 ml fresh distilled aniline for 18 h at 403 K. After reaction completed (TLC control) was added 50 ml e thanol, at room temperature. The brown precipitate was sucked, washed with cold ethanol(2*15 ml), dried over sodium sulfateand gave 3.3 g(63%) (Schelz & Inst,1978). Pure compound (I) was obstained by crystallizing from ethanol. Crystals of (I) suitable for X-ray diffraction were obstained by slow evaporation of an ethanol solution.

### Refinement

H atoms were positioned geometrically, with C—H = 0.93 Å for aromatic H, and constrained to ride on their parent atoms, with *U*_iso_(H) = 1.2*U*_eq_(C).

### Crystal data

**Table:**

C_13_H_9_N_3_O_2_	*F*(000) = 496
*M**_r_* = 239.23	*D*_x_ = 1.381 Mg m^−^^3^
Monoclinic, *P*2_1_/*c*	Mo *K*α radiation, λ = 0.71073 Å
Hall symbol: -P 2ybc	Cell parameters from 25 reflections
*a* = 14.066 (3) Å	θ = 9–13°
*b* = 7.4290 (15) Å	µ = 0.10 mm^−^^1^
*c* = 11.652 (2) Å	*T* = 293 K
β = 109.04 (3)°	Block, colourless
*V* = 1151.0 (4) Å^3^	0.30 × 0.30 × 0.10 mm
*Z* = 4	

### Data collection

**Table:**

Enraf–Nonius CAD-4 diffractometer	1546 reflections with *I* > 2σ(*I*)
Radiation source: fine-focus sealed tube	*R*_int_ = 0.030
graphite	θ_max_ = 25.3°, θ_min_ = 1.5°
ω/2θ scans	*h* = −16→15
Absorption correction: ψ scan (North *et al.*, 1968)	*k* = −8→8
*T*_min_ = 0.972, *T*_max_ = 0.990	*l* = 0→13
4199 measured reflections	3 standard reflections every 200 reflections
2082 independent reflections	intensity decay: 1%

### Refinement

**Table:**

Refinement on *F*^2^	Primary atom site location: structure-invariant direct methods
Least-squares matrix: full	Secondary atom site location: difference Fourier map
*R*[*F*^2^ > 2σ(*F*^2^)] = 0.040	Hydrogen site location: inferred from neighbouring sites
*wR*(*F*^2^) = 0.107	H-atom parameters constrained
*S* = 1.04	*w* = 1/[σ^2^(*F*_o_^2^) + (0.0511*P*)^2^ + 0.1451*P*] where *P* = (*F*_o_^2^ + 2*F*_c_^2^)/3
2082 reflections	(Δ/σ)_max_ < 0.001
163 parameters	Δρ_max_ = 0.19 e Å^−^^3^
0 restraints	Δρ_min_ = −0.16 e Å^−^^3^

### Special details

**Table:**

Geometry. All esds (except the esd in the dihedral angle between two l.s. planes) are estimated using the full covariance matrix. The cell esds are taken into account individually in the estimation of esds in distances, angles and torsion angles; correlations between esds in cell parameters are only used when they are defined by crystal symmetry. An approximate (isotropic) treatment of cell esds is used for estimating esds involving l.s. planes.
Refinement. Refinement of *F*^2^ against ALL reflections. The weighted *R*-factor *wR* and goodness of fit *S* are based on *F*^2^, conventional *R*-factors *R* are based on *F*, with *F* set to zero for negative *F*^2^. The threshold expression of *F*^2^ > σ(*F*^2^) is used only for calculating *R*-factors(gt) *etc*. and is not relevant to the choice of reflections for refinement. *R*-factors based on *F*^2^ are statistically about twice as large as those based on *F*, and *R*- factors based on ALL data will be even larger.

### Fractional atomic coordinates and isotropic or equivalent isotropic displacement parameters (Å^2^)

**Table:**

	*x*	*y*	*z*	*U*_iso_*/*U*_eq_	
O1	0.91767 (8)	0.4271 (2)	0.39718 (10)	0.0665 (4)	
O2	0.84253 (9)	0.43363 (19)	0.20463 (10)	0.0659 (4)	
N1	0.82164 (10)	0.5168 (2)	0.54802 (11)	0.0545 (4)	
H1	0.8774	0.4845	0.5388	0.065*	
N2	0.84229 (10)	0.45587 (19)	0.30832 (11)	0.0485 (4)	
N3	0.43509 (11)	0.6904 (2)	0.03546 (14)	0.0699 (5)	
C1	0.74708 (13)	0.4539 (2)	0.70743 (15)	0.0570 (5)	
H1B	0.6921	0.3979	0.6517	0.068*	
C2	0.75504 (15)	0.4625 (3)	0.82822 (17)	0.0669 (5)	
H2A	0.7042	0.4143	0.8535	0.080*	
C3	0.83667 (16)	0.5411 (3)	0.91186 (16)	0.0695 (6)	
H3A	0.8414	0.5451	0.9933	0.083*	
C4	0.91129 (15)	0.6136 (3)	0.87473 (16)	0.0656 (5)	
H4A	0.9671	0.6659	0.9314	0.079*	
C5	0.90432 (12)	0.6097 (3)	0.75389 (14)	0.0537 (4)	
H5A	0.9548	0.6605	0.7290	0.064*	
C6	0.82202 (11)	0.5299 (2)	0.67003 (13)	0.0463 (4)	
C7	0.74482 (11)	0.5487 (2)	0.44502 (13)	0.0438 (4)	
C8	0.65218 (12)	0.6207 (2)	0.44808 (14)	0.0489 (4)	
H8A	0.6447	0.6444	0.5230	0.059*	
C9	0.57426 (12)	0.6561 (2)	0.34586 (14)	0.0506 (4)	
H9A	0.5150	0.7039	0.3520	0.061*	
C10	0.58206 (11)	0.6214 (2)	0.23110 (14)	0.0461 (4)	
C11	0.67092 (11)	0.5533 (2)	0.22313 (13)	0.0455 (4)	
H11A	0.6771	0.5300	0.1474	0.055*	
C12	0.75095 (11)	0.5196 (2)	0.32739 (13)	0.0432 (4)	
C13	0.49993 (12)	0.6596 (2)	0.12224 (15)	0.0524 (4)	

### Atomic displacement parameters (Å^2^)

**Table:**

	*U*^11^	*U*^22^	*U*^33^	*U*^12^	*U*^13^	*U*^23^
O1	0.0436 (7)	0.1033 (11)	0.0496 (7)	0.0174 (7)	0.0112 (5)	−0.0019 (6)
O2	0.0553 (7)	0.1003 (11)	0.0471 (7)	0.0071 (7)	0.0237 (6)	−0.0053 (6)
N1	0.0437 (7)	0.0796 (11)	0.0403 (7)	0.0117 (7)	0.0140 (6)	0.0013 (7)
N2	0.0431 (7)	0.0617 (9)	0.0421 (7)	0.0019 (7)	0.0157 (6)	−0.0028 (6)
N3	0.0545 (9)	0.0920 (13)	0.0557 (9)	0.0140 (9)	0.0076 (8)	0.0037 (9)
C1	0.0556 (10)	0.0621 (11)	0.0535 (10)	−0.0002 (9)	0.0182 (8)	0.0053 (9)
C2	0.0700 (12)	0.0752 (13)	0.0661 (12)	0.0131 (11)	0.0369 (10)	0.0185 (10)
C3	0.0884 (15)	0.0803 (14)	0.0438 (10)	0.0311 (12)	0.0268 (10)	0.0111 (9)
C4	0.0634 (11)	0.0753 (13)	0.0466 (10)	0.0174 (10)	0.0022 (9)	−0.0035 (9)
C5	0.0428 (9)	0.0686 (11)	0.0476 (9)	0.0092 (8)	0.0120 (7)	0.0026 (8)
C6	0.0449 (8)	0.0540 (10)	0.0394 (8)	0.0119 (8)	0.0130 (7)	0.0038 (7)
C7	0.0430 (8)	0.0468 (9)	0.0418 (8)	−0.0007 (7)	0.0140 (7)	−0.0001 (7)
C8	0.0485 (9)	0.0578 (10)	0.0431 (9)	0.0042 (8)	0.0189 (7)	−0.0009 (8)
C9	0.0431 (9)	0.0561 (10)	0.0543 (10)	0.0058 (8)	0.0181 (8)	0.0022 (8)
C10	0.0395 (8)	0.0509 (10)	0.0450 (9)	−0.0007 (7)	0.0102 (7)	0.0020 (7)
C11	0.0461 (9)	0.0509 (10)	0.0394 (8)	−0.0027 (7)	0.0140 (7)	−0.0013 (7)
C12	0.0389 (8)	0.0472 (9)	0.0442 (8)	0.0013 (7)	0.0144 (7)	−0.0013 (7)
C13	0.0449 (9)	0.0615 (11)	0.0502 (9)	0.0041 (8)	0.0148 (8)	0.0021 (8)

### Geometric parameters (Å, °)

**Table:**

O1—N2	1.2352 (16)	C4—C5	1.379 (2)
O2—N2	1.2206 (16)	C4—H4A	0.9300
N1—C7	1.3483 (18)	C5—C6	1.382 (2)
N1—C6	1.4232 (19)	C5—H5A	0.9300
N1—H1	0.8600	C7—C12	1.418 (2)
N2—C12	1.4529 (19)	C7—C8	1.420 (2)
N3—C13	1.142 (2)	C8—C9	1.356 (2)
C1—C2	1.377 (2)	C8—H8A	0.9300
C1—C6	1.385 (2)	C9—C10	1.401 (2)
C1—H1B	0.9300	C9—H9A	0.9300
C2—C3	1.371 (3)	C10—C11	1.379 (2)
C2—H2A	0.9300	C10—C13	1.438 (2)
C3—C4	1.369 (3)	C11—C12	1.383 (2)
C3—H3A	0.9300	C11—H11A	0.9300
			
C7—N1—C6	128.16 (14)	C5—C6—N1	117.71 (15)
C7—N1—H1	115.9	C1—C6—N1	122.08 (15)
C6—N1—H1	115.9	N1—C7—C12	123.37 (14)
O2—N2—O1	121.83 (13)	N1—C7—C8	121.29 (14)
O2—N2—C12	118.93 (13)	C12—C7—C8	115.30 (14)
O1—N2—C12	119.24 (13)	C9—C8—C7	122.46 (15)
C2—C1—C6	119.09 (17)	C9—C8—H8A	118.8
C2—C1—H1B	120.5	C7—C8—H8A	118.8
C6—C1—H1B	120.5	C8—C9—C10	120.70 (15)
C3—C2—C1	121.11 (18)	C8—C9—H9A	119.7
C3—C2—H2A	119.4	C10—C9—H9A	119.7
C1—C2—H2A	119.4	C11—C10—C9	119.11 (14)
C4—C3—C2	119.57 (17)	C11—C10—C13	119.86 (15)
C4—C3—H3A	120.2	C9—C10—C13	121.01 (15)
C2—C3—H3A	120.2	C10—C11—C12	120.19 (15)
C3—C4—C5	120.51 (18)	C10—C11—H11A	119.9
C3—C4—H4A	119.7	C12—C11—H11A	119.9
C5—C4—H4A	119.7	C11—C12—C7	122.20 (14)
C4—C5—C6	119.68 (18)	C11—C12—N2	115.57 (13)
C4—C5—H5A	120.2	C7—C12—N2	122.22 (13)
C6—C5—H5A	120.2	N3—C13—C10	179.6 (2)
C5—C6—C1	120.02 (15)		
			
C6—C1—C2—C3	−1.5 (3)	C8—C9—C10—C11	−1.1 (3)
C1—C2—C3—C4	0.5 (3)	C8—C9—C10—C13	−179.63 (16)
C2—C3—C4—C5	0.7 (3)	C9—C10—C11—C12	0.1 (2)
C3—C4—C5—C6	−0.8 (3)	C13—C10—C11—C12	178.70 (15)
C4—C5—C6—C1	−0.1 (3)	C10—C11—C12—C7	1.5 (2)
C4—C5—C6—N1	−175.33 (16)	C10—C11—C12—N2	−177.32 (14)
C2—C1—C6—C5	1.3 (3)	N1—C7—C12—C11	179.94 (15)
C2—C1—C6—N1	176.26 (16)	C8—C7—C12—C11	−2.1 (2)
C7—N1—C6—C5	−137.13 (18)	N1—C7—C12—N2	−1.3 (2)
C7—N1—C6—C1	47.8 (3)	C8—C7—C12—N2	176.63 (15)
C6—N1—C7—C12	−175.60 (16)	O2—N2—C12—C11	−1.2 (2)
C6—N1—C7—C8	6.6 (3)	O1—N2—C12—C11	178.27 (15)
N1—C7—C8—C9	179.15 (16)	O2—N2—C12—C7	180.00 (15)
C12—C7—C8—C9	1.2 (2)	O1—N2—C12—C7	−0.6 (2)
C7—C8—C9—C10	0.4 (3)		

### Hydrogen-bond geometry (Å, °)

**Table:**

*D*—H···*A*	*D*—H	H···*A*	*D*···*A*	*D*—H···*A*
N1—H1···O1	0.86	1.96	2.6280 (18)	134
C3—H3A···O2^i^	0.93	2.59	3.478 (2)	159

Symmetry codes: (i) *x*, *y*, *z*+1.
